# A241 CHARACTERIZING KUPFFER CELL-VIRUS INTERACTIONS IMMEDIATELY FOLLOWING VIRAL INVASION AND IN ACUTE HEPATITIS IN THE WOODCHUCK HEPATITIS VIRUS MODEL OF HEPATITIS B VIRUS INFECTIONS

**DOI:** 10.1093/jcag/gwac036.241

**Published:** 2023-03-07

**Authors:** L Al-Yasiri, R P Davis, S D'souza, T R Patel, F van der Meer, D P Whiteside, T I Michalak, C N Jenne, C S Coffin

**Affiliations:** 1 Microbiology and Infectious Diseases; 2 Immunology, University of Calgary, Calgary; 3 Chemistry and Biochemistry, University of Lethbridge, Lethbridge; 4 Faculty of Veterinary Medicine, University of Calgary, Calgary; 5 Molecular Virology & Hepatology Research Group, Memorial University of Newfoundland, St. John's; 6 Microbiology, Immunology and Infectious Diseases; 7 Department of Medicine, University of Calgary, Calgary, Canada

## Abstract

**Background:**

Hepatitis B virus (HBV) infections are one of the largest global infectious disease burdens, despite the availability of a preventative vaccine. The virus infects the liver which is a vital organ that plays a critical role in homeostasis due to its metabolic and immunoregulatory functions. Kupffer cells (KCs) are considered to be a first line of immune defense for protection of hepatocytes. Both woodchuck hepatitis virus (WHV) and HBV are members of *Hepadnaviridae* and demonstrate comparable infection outcomes and progression to hepatocellular carcinoma. North American woodchucks (*Marmota monax*) infected with WHV present a natural infection model for the assessment of acute and chronic hepadnaviral infections. We hypothesize that KC absence in acute viral hepatitis leads to chronic infections by impacting downstream virus processing and immune responses.

**Purpose:**

We aim to uncover the early host-virus interactions in acute WHV infections to facilitate our understanding of the risk of progression to chronic infection and the immunopathogenic mechanisms involved in chronic hepatitis.

**Method:**

We designed and optimized in-house WHV and woodchuck-specific assays to assess intrahepatic and systemic virus presence and anti-viral immune responses which occur following WHV invasion (<24 hours) and infection (4-6 weeks). A live imaging protocol was developed for capturing real-time events in a living host. Next, we optimized macrophage depletion in woodchucks to evaluate virus invasion and acute infection outcomes in the presence and absence of KCs. Using purified and fluorescently labelled WHV virions (concentrations exceeding 10^10^VGE copies/ml), we imaged host cell-hepadnaviral interactions and characterized immunological and virological differences between KC-depleted animals and non-depleted animals.

**Result(s):**

*Ex vivo *assessment of the consequences of KC depletion during invasion have been evaluated within 1- and at 24-hour post-virus injection. Intravital microscopy (IVM) revealed that the virus traffics to, and accumulates within, the liver 10 seconds following intravenous administration, demonstrating direct binding and capture by liver macrophages. In contrast, virus only begins to accumulate in the KC-non-depleted control minutes after infection in a randomized manner. Preliminary invasion results indicate that in both the KC-depleted and non-depleted animals, virus is most readily detectable in the blood within the first hour following viral administration. By 24-hours post-virus injection the highest viral load in **non-depleted woodchucks **was identified in the liver whereas in **KC-depleted woodchucks, **the highest viral loads were found in the spleen 24-hours post-injection.

**Conclusion(s):**

We have successfully captured the early host-virus interactions and continue to uncover the role of KCs in viral hepatitis infections. Tracking the initial interactions allows us to understand the events promoting viral dissemination and development of chronic hepatitis.

**Disclosure of Interest:**

None Declared

